# Recent Nanotechnological Trends in the Management of Microbial Keratitis

**DOI:** 10.18502/jovr.v19i4.14498

**Published:** 2024-12-31

**Authors:** Shraddha Jaiswal, Prabhavati Shinde, Vidya Tale

**Affiliations:** ^1^Rajiv Gandhi Institute of IT And Biotechnology, Bharati Vidyapeeth Deemed to be University, Pune, India; ^3^Vidya Tale: https://orcid.org/0000-0002-7883-8044; ^4^Shraddha Jaiswal: https://orcid.org/0000-0003-4281-9631

**Keywords:** Antimicrobial Resistance, Contact Lenses, Corneal Diseases, Microbial Keratitis, Nanotechnology

## Abstract

Microbial keratitis (MK) is a sight-threatening ocular disease that needs rapid diagnosis and treatment to prevent more serious outcomes. The broad-spectrum topical antimicrobial treatment is currently the main pharmacological approach for MK management, yet its efficacy is increasingly challenged by evolving antimicrobial resistance, including multidrug resistance. Also, the ocular surface presents numerous challenges for standard topical drug delivery. The failure and ineffectiveness of current therapies have necessitated the development of novel therapeutic strategies to manage MK. With advances in nanotechnology in the biomedical field, various nanomaterials can be employed to control MK. The primary determinants of nanoparticles' vast range of applications are their size, surface properties, and chemical makeup, which also happen to be the same elements that give rise to their poisonous and dangerous effects. In this study, we provide a perspective on the contact lens-associated corneal illnesses such as MK and explore how nanotechnology might help address this significant clinical issue. In addition, safety and toxicological concerns about the increasingly widespread use of contact lenses are also discussed.

##  INTRODUCTION

Microbial Keratitis (MK) is an eye infection characterized by excruciating eye pain, blurred vision, corneal ulceration, and stromal infiltrates. It is the most common reason for corneal blindness in both developed and developing countries.^[1]^ MK could be caused by a wide range of infectious agents, including bacteria, fungi, protozoa, and viruses. Furthermore, polymicrobial infection has been linked to 2–15% of all MK cases. Since the ocular surface is equipped with highly regulated innate and adaptive defensive systems, MK is uncommon in the absence of predisposing circumstances such as contact lens (CL) wear, trauma], ocular surface disorders, and post-corneal surgery.^[2,3,4]^

MK is often treated with conventional medications; however, due to poor ocular bioavailability, short ocular residence time, high rates of antibiotic resistance, and occasional adverse responses, the traditional drug delivery strategy has not been able to treat the condition completely. As a result, researchers are now focused on identifying an effective delivery method for more efficacious ophthalmic drugs, such as one that uses lipid and polymeric nanoparticles (NPs).^[5,6]^

Although the subject has been covered in several works before,^[1,4,6,7,8,9,10]^ this narrative review primarily discusses the epidemiology of MK and its risk factors in addition to recent studies (mostly between 2015 and 2023) on the use of nanotechnology-based tools for MK management. These tools, when tested against infections causing microorganisms, have shown encouraging results that highlight their usefulness and adaptability.

##  METHODS

A literature search was performed in PubMed, Medline Plus, and Google Scholar using a combination of the following keywords: “Ocular infection”, “Microbial keratitis”, “Ocular drug delivery”, “Nanoparticles”, “Nanocarriers”, “Nanomedicine”, and “Contact lens”. In addition, we used the “Ocular infection” MeSH term. One keyword/phrase from each cluster was used without repetition. All case reports, open-access reviews, and original articles published in English until May 2023 were screened and assessed and then incorporated into our analysis. References from selected articles and relevant review articles were manually searched and included in this review, as appropriate.

### Contact lens related microbial keratitis

MK is a major health issue affecting the cornea and it is the fifth most common cause of blindness worldwide.^[1]^ Wearing CLs contributes to over half of bacterial keratitis cases.^[11]^ Risk factors include rigid gas-permeable lenses, daily disposable lenses, and periodically replaced lenses. Younger individuals are more prone to developing MK. Participation in water sports, showering while wearing lenses, and sleeping while wearing lenses are common risk factors.^[2]^ Over 85% of cases of acanthamoeba keratitis (AK) occur in CL wearers, with water exposure being the main risk factor.^[12]^ Poor personal hygiene, overnight wear of CLs, prolonged storage of CLs in contaminated solutions (such as tap water or preservative-free saline solution), and reusing CL solutions can increase the risk of MK.^[8,12,13,14]^ A significant risk factor of CL-associated Fusarium keratitis in 2005–2006 was attributed to the growth of Fusarium biofilm on CLs.^[15]^

### Pathogenesis of microbial keratitis

According to the results of the systematic review by Hatami et al, the three most common organisms associated with CL-related bacterial keratitis were *Pseudomonas aeruginosa*, *Staphylococcus species*, and *Serratia marcescens* in resource-limited regions like South America, Asia, and Africa.^[7,9]^ *Corynebacterium *species*, Haemophilus influenzae, Propionibacterium acnes, *and *Serratia marcescens* were the less common bacterial isolates. The degree of keratitis at presentation varied greatly depending on the culture result. Patients with positive cultures for *Fusarium,* ^[16]^ *P. aeruginosa*, and other Gram-negative organisms presented severe keratitis, compared to patients with negative cultures.^[4]^ Fungal keratitis, primarily caused by *Fusarium* and *Aspergillus *genera, is an emerging global health concern. The prevalence of fungal keratitis is increasing due to the use of extended-wear CLs and outdoor activities, which can cause corneal trauma.^[15]^

### Conventional therapy and limitation

The gold standard treatment for ocular infections (including MK) is broad-spectrum topical antibiotic medication, either in the form of fluoroquinolone monotherapy or a combination of cephalosporin and aminoglycoside. Patients are treated for fungal and bacterial keratitis using topical eye drops and antibacterial medications. Natamycin 5% and fluconazole are used to treat fungal keratitis, whereas moxifloxacin 0.5% eye drops are used to treat secondary infections. Deep ulcers are generally treated with itraconazole. Treatments are tailored to culture results, with vancomycin being particularly effective against *Pseudomonas aeruginosa*. For severe cases of diffuse fungal keratitis, surgical debridement and keratoplasty are performed and evisceration may even be suggested.^[17]^

Antibiotic therapy is the most common therapeutic method for treating ophthalmic infections, but it can be limited due to antibiotic resistance and possible side effects such as chemosis, hyperemia, corneal precipitations, and allergic reactions. Additionally, conventional ophthalmic antibiotics have a short ocular residence length, reduced corneal penetration, limited bioavailability, and poor administration.^[5]^

Recent studies on MK have stressed the rise in antimicrobial resistance (AMR) in eye infections, particularly in the United States,^[18]^ China,^[19]^ and India.^[20]^ Possible causes include inappropriate use of antibiotics in both ocular and systemic diseases, improper dosing, and drug resistance. There is a growing number of reports assessing antibiotic sensitivity and resistance of MK-related bacteria. Geographical conditions and temporal factors result in different AMR patterns in eye infections. A study conducted in Southern China revealed that multidrug resistance was commonly observed in *S. pneumoniae*, *S. epidermidis*, *S. aureus*, and *P. aeruginosa*.^[19]^ The fourth-generation fluoroquinolone gatifloxacin was approved for treatment as a monotherapy in gram-negative-related MK, since it was found to be 90% effective against *P. aeruginosa* and *Acinetobacter species*. According to a report from South China, the emergence of methicillin-resistant bacteria increased from 2010 to 2018, but susceptibility to fluoroquinolone and aminoglycoside remained unchanged.^[21]^ A study in Northern India found a significant proportion of *P. aeruginosa* strains to be resistant to ciprofloxacin, moxifloxacin, and aminoglycoside, emphasizing the geographic variation in AMR patterns and the necessity of region-specific investigations into AMR profiles in ocular infections.^[20,22]^ A study conducted in the United States discovered a significant rate of AMR, specifically methicillin resistance, among *Staphylococci *and *Streptococci species*. The authors added that this risk would increase with age. According to a Mexican study, 21–79% of *S. aureus* and 48–71% of *CoNS* were resistant to oxacillin, while *P. aeruginosa* and other gram-negative infections were resistant to oxacillin and vancomycin.^[21]^

Several studies over the past 10 years have noted an increase in eye infections associated with methicillin-resistant *Staphylococcus aureus* (MRSA). Also, an emerging trend of AMR, especially methicillin resistance, was found among *Staphylococci *and *Streptococci species*, according to a US study on antibiotic resistance among ocular bacteria. It was found that the risk increased with age and that 75% of MRSA and MR-*CoNS* were resistant to multiple drugs.^[22]^

The increasing prevalence of AMR in eye infections is a major concern, highlighting the need for region-specific research on AMR patterns in eye infections. To overcome these inadequacies, it appears that alternative therapeutic strategies must be developed. Recently, it has been proposed that eye drugs be synthesized in nano-drug delivery systems for sustained and effective therapy.^[8]^

### Nanotechnology as a tool for the management of microbial keratitis

It is anticipated that strategies based on nanotechnology would offer encouraging improvements in combatting drug-resistant biofilm infections of medical devices and biomaterials.^[1]^ The usage of NP-coated surfaces as biofilm-reducing agents has been documented in several studies.^[23,24,25]^ Materials exhibit distinct physical, chemical, and biological characteristics at the nanoscale, as well as potentially other phenomena like quantum effects that cannot be observed in their bulk counterparts. Nanomaterials resemble biomolecules in size and have significantly higher surface area-to-volume ratios, which lead to enhanced chemical and biological reactivities. Additionally, because NPs are extremely small, they can pass through biofilm layers and microbial cell walls, which can permanently harm DNA and cell membranes. Additionally, their high surface-to-volume ratios and prolonged plasma half-lives make it easier to load medicines and target molecules on them.^[6]^

**Figure 1:**
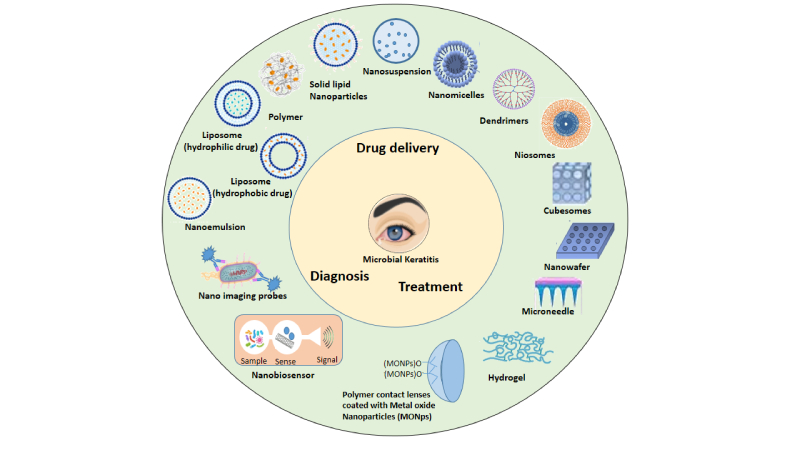
Novel nanotechnological approach for the detection and management of infectious microbial keratitis.

Nanotechnology has enabled the development of new strategies for the formulation of ocular drugs. Lipid and polymeric NPs ranging from 1 to 100 nanometers (nm) in size have been found to exhibit greater permeability through biological membranes, hence increasing the drug's bioavailability and ocular residence time. As a result, lipid and polymeric NPs have recently been used to enhance the formulation of numerous medications for greater corneal penetration and residence duration.^[26]^ Figure 1 illustrates MK management using nanomaterials.

### Nanotechnology for pathogen detection

Early detection of microbe types is critical for controlling bacterial eye infections. Traditional testing procedures are time-consuming and may not capture optimal treatment times. The distinctive anatomy of the eye limits the number of specimens available for analysis, possibly reducing detection rates.^[27]^

Nanotechnology is now utilized to detect pathogens through optical, magnetic, or electrochemical approaches that exploit the unique properties of nanomaterials. For instance, controllable optical characteristics enable identifying infectious microorganisms via fluorescence, colorimetry, and surface-enhanced Raman spectroscopy. The magnetic properties make it easy to isolate, enrich, and purify harmful bacteria, leading to faster detection times. The distinctive electrochemical properties provide opportunities for sensing and recognition. Thus, this nano imaging and nano biosensing technology can be exploited for rapid detection of pathogen.^[28]^

Zhang et al developed a noninvasive, fast in situ imaging system using a fluorescent silicon nanoparticle connected to vancomycin (SiNPs-Van), which detected keratitis caused by gram-positive bacteria.^[29]^ Similarly, Zhao et al created a silica NP coated with vancomycin-modified polyelectrolyte-cypate (Van-SiO2/polyelectrolyte-Cy) complex nanosystem. In this system, the hydrophobic Cy fluorophore aggregates on SiO ${\;}_{2}$ and remains non fluorescent in the absence of MRSA. On the other hand, in the presence of MRSA, vancomycin and polyelectrolyte-Cy complex dissociates from SiO ${\;}_{2}$ and binds it on MRSA bacterial cell surface. Consequently, the fluorescence of the Cy fluorophore changes from an “off” state (bound to SiO ${\;}_{2}$ ) into an "on" state (dissociated and bound to the bacteria).^[30]^

Capeletti et al created glucosamine-functionalized SiO ${\;}_{2}$ NPs (glc-SiO2 NPs) as a lipopolysaccharide (LPS) targeting probe that identifies bacteria and inhibits NP aggregation and adsorption of protein.^[31]^

The unique chemical, physical, sensory, and photosensitive properties of nanomaterials such as metal oxide NPs, metal NPs, carbon NPs, and quantum dots (QDs) can be harnessed to develop biosensors for bacterial detection. Qi et al suggested a rapid bacterial detection sensor based on multivalent glycosylated copper doped cadmium sulfide QDs; the sensor managed to identify six bacterial strains with more than 90% accuracy.^[32]^ Similarly, Zheng et al showed a novel fluorescence sensor array using cadmium, tagged with three receptors (boric acid, polymyxin, and vancomycin). This fluorescent sensor array allows for rapid and accurate recognition of six different bacterial species.^[33]^

Wang et al proposed a colorimetric detection method, whereby hydrogel is prepared by incorporating bromothymol blue (BTB). BTB is a pH-sensitive sensitive dye that changes color from blue to yellow in acidic microenvironment due to changes in its chemical structure. This hydrogel can sense S. aureus infections based on the visible change in color.^[34]^

### Nanotechnology for drug delivery

By enabling controlled drug release, minimizing eye irritation, reinforcing ocular tissue compatibility, and improving medication absorption, nanotechnology has opened up new possibilities in the therapy of ocular illnesses in recent decades.^[18]^ Different nanosystems can deliver their payloads to the posterior and anterior chambers of the eye. Multiple NPs have recently been produced as effective carriers for different antimicrobial medicines, suggesting potential for enhanced treatment of various infections.^[35]^

In recent years, nanomaterials such as metallic NPs, metal-oxide NPs, lipid-based NPs, and polymeric NPs have emerged as promising candidates for use as drug delivery vehicles.^[6]^ Natural or manufactured polymeric materials are the basic building blocks of these nanosystems. Many colloidal systems fall under this category, including cyclodextrins, liposomes, niosomes, dendrimers, *in situ* hydrogels, and micelles.^[36]^

Organic NPs have also gained attention for their potential medical applications. Liposomes are the first nanoscale medication approved for medical use, and they are a biocompatible, biodegradable, and nontoxic vesicle with an efficient drug-loading capacity. Of note, encapsulating hydrophilic antimicrobial drugs in a liposomal NP core can protect them from degradation *in vivo*.

Studies have shown the curative effect of polymer-based NPs, such as polylactic-co-glycolic acid, dendrimers, and micelles, which have unique structural properties for drug delivery.^[35]^ Owing to their unique physicochemical characteristics at the nanoscale, dendrimers are capable of encapsulating drugs through different mechanisms, such as hydrogen bonding, ionic interactions, and hydrophobic forces. Polyamide-amine (PAMAM) is a major class of dendrimer materials that attaches to corneal epithelium by interacting with the mucin layer, thereby connecting with the lipid bilayer of cells of corneal epithelium. Dendritic macromolecules serve as antibacterial medication transporters while also exhibiting antimicrobial and anti-biofilm properties.^[37]^ Dhumal et al demonstrated the novel self-assembly of supramolecular dendritic nanosystems. In this process, electrostatic interactions concentrate dendritic molecules on the cell surface of the bacteria, which then self-assemble into supramolecular nanocomponents. The hydrophobic tail of dendritic molecules penetrates the bacterial cell membrane, resulting in lysis.^[38]^

Microneedle for ocular bacterial infections reduces discomfort and delivers antimicrobial drugs at the site of infection. Currently, three types of microneedles are employed to administer ocular drugs: coated, hollow, and soluble.^[39]^ Park et al integrated silicon nanoneedles (Si NNs) with a tear-soluble CL to improve ocular drug penetration. This modified CL accurately fits the cornea and serves as a temporary scaffold that disintegrates quickly and is cleared by tears within 60 seconds, allowing patients to preserve clear vision. Si NNs function well on tear-soluble CLs, suggesting potential treatment of persistent infections of the cornea.^[39]^

Inorganic NPs are classified into metal NPs and metal oxide NPs, each with unique antibacterial properties. These NPs can be loaded with antibiotics, exerting a dual mechanism of action. Metal NPs include silver (Ag),^[40]^ gold (Au),^[41]^ and copper (Cu);^[42]^ non-metal NPs include silica (Si),^[43]^ nickel (Ni), and selenium (Se);^[35]^ and metal oxide NPs include zinc oxide (ZnO),^[24]^ titanium dioxide (TiO ${\;}_{2}$ ),^[44]^ and copper oxide (CuO)^[45]^ among others. Organic NPs have greater surface area-to-volume ratios but have lower biodegradability and biocompatibility.^[5]^ Inorganic NPs may show cytotoxicity, depending on their size, charge, and dosage.^[46]^ Carbon nanotubes are a promising alternative in drug delivery due to their unique biological, physical, and chemical properties. Mesoporous silica NPs are promising drug delivery carriers that possess a greater surface area and sturdy framework. Besides, their porous nature allows them to encapsulate a high number of antimicrobial agents.^[6]^

Hybrid NPs are composite drug delivery systems that combine organic and inorganic NPs, improving biological efficacy and reducing toxicity and resistance. These hybrid lipid polymer NPs are effective as they are biocompatible and offer sustained drug release. They can be loaded with hydrophilic and hydrophobic drugs, achieving perfect drug targeting and release.^[47]^ Liposomes can be replaced by nanostructured lipid carriers and solid lipid NPs (SLN) for drug delivery. Lipid NPs are more cost-effective and have lower medication leakage than liposomes. To treat bacterial keratitis, SLNs are often modified with polyethylene glycol or chitosan to improve their pharmacokinetics and extend residence time in the cornea. Furthermore, PEG-tagged SLNs enhance drug loading in CLs and exhibit sustained drug release.^[27]^

Chitosan is a biocompatible and biodegradable material that is capable of reducing protein deposition on surfaces. Voriconazole is a broad-spectrum antifungal drug employed in ophthalmology for treatment of fungal infection. Niu et al developed a glycol chitosan-based nanodrug that integrated 4-carboxyphenylboronic acid pinacol (EB) and voriconazole to treat fungal keratitis. *In vitro* and *in vivo* studies confirmed effective and successful delivery and penetration of Chitosan-based nanodrug in the cornea followed by reduced oxidative stress and inflammation, demonstrating therapeutic action against mycotic keratitis.^[48]^ In another study, fluconazole, a broad-spectrum antifungal drug, was encapsulated within liposomes using the reverse-phase evaporation technique. The purpose was to achieve extended drug delivery time and rapid therapeutic action in mouse models of Candida keratitis and to compare its efficacy with the efficacy of fluconazole alone.^[49]^

Hydrogel NPs, also known as polymeric nanogels or macromolecular micelles, are a potential therapeutic drug carrier. These nanostructures are adaptable and have appropriate properties for biopharmaceutical delivery of bioactive compounds. Stimuli-responsive hydrogels, including pH-, thermo-, and ion-sensitive hydrogels, are now a promising area of research.^[6]^ Cheng et al created a hydrogel composed of guanosine 5'-monophosphate disodium salt (GMP) and tobramycin. The GMP nanofibers in this hydrogel may be formed into G-quadruplex nanofibers, generating a gel that can be used to clinically treat bacterial keratitis.

### Nanotechnology for the development of antimicrobial contact lenses and lens cases to prevent microbial keratitis

CLs and their cases are often infected by gram-negative bacteria, which can cause corneal ulcers. According to a study, in 25% of cases, the organisms were isolated from CLs and their storage cases, isolating organisms from these sources may be easier than corneal scrapings.^[36]^ However, the identification of organisms isolated from CLs and cases cannot be a reliable guide for antibiotic treatment.^[36]^

In recent decades, the nanomaterial coating technique has been explored in CL production by coating the lens with a multifunctional substance to promote hydration and antimicrobial activity. There are various methods for incorporating antibiotics in CLs.^[50]^ One method involves drug loading by dispersing the drug in varying concentrations and then mixing it with a monomer or polymer system.^[51]^ Another method is to add suitable surfactants to the monomer and then polymerizing it along with the drug or NP. Another way for coating CLs is to immerse them directly into the drug-loaded NPs suspension.^[42]^

CLs might be coated with copper,^[52]^ zinc NPs,^[53]^ and zinc doped copper oxide^[54]^ nanomaterials. Selenium-based nano coatings on CLs and lens covers serve the same purpose. Zwitterionic polymeric nano coatings on CLs can electrostatically interact with the aqueous environment of the eye, promoting steady hydration. Some authors have used hydrophilic polymers to functionalize the surface of CLs, and they have reported improved wettability.^[55]^

Chitosan nanocomposite materials are widely used to manufacture CL surfaces. Electrohydrodynamic atomization is a recent development for applying nanomaterial coatings to the surface of CLs.^[56]^ This method has been used for controlled drug release, such as timolol maleate using chitosan as a nanocarrier, and the results have supported its efficacy in treating glaucoma.^[57]^ Chitosan nanocomposite materials increase the penetration of the drug due to their mucoadhesive properties. Chitosan is compatible with the ocular tissue and its cationic property facilitates drug transport through interactions with the cell membrane.^[56]^

Garhwal et al reported the development of ciprofloxacin-loaded core-shell micellar nanospheres for application in CLs, and the results demonstrated controlled, sustainable, and effective drug delivery for ocular illnesses.^[58]^

Silver NPs have been studied extensively, and silver-coated lens cases are now commercially accessible. However, silver NPs are not employed directly in CLs because they are found to interfere with the proliferation of corneal cells. Wenwen Qu et al showed that bacterial infection is more likely to occur in Ag-impregnated lens cases than in polypropylene lens cases as adhesion forces of bacteria are reduced on lens case but this effect is counteracted by increased bacterial decontamination as a response to Ag impregnation, especially when silver-impregnated lens cases are used in conjunction with antimicrobial lens care solutions. This finding highlights the need to consider not only antibacterial lens care products but also the surface qualities of a CL and its case.^[59]^

A new and successful method for treating many ocular illnesses and overcoming the limitations of regular eye drops is the use of CLs as a platform for ocular medication delivery. To ensure consumer safety and comfort, further research must be done before the widespread adoption of CLs containing antimicrobial agents.^[60]^ The studies analyzed in this review [Table 1] maximize the role of nanomaterial in the inhibition of infectious agents associated with CLs.

Several studies recommend nanosystems for administering ocular medications, however, additional research is necessary to understand the penetration and mucoadhesive mechanisms between NPs and the corneal barrier.^[1]^ It has been demonstrated that the epithelium of the cornea acts as a primary barrier to penetration and permeation, preventing particles even < 20 nm from entering the intraocular space.^[61]^ CLs containing NPs are a type of polymeric nanodevice that can carry pharmaceutical agents in hydrogel and offer sustained drug release on the corneal surface, which can be beneficial for CL wearers.^[62]^

### Nanotechnology for the development of an antimicrobial media for soft contact lens storage

Corneal infection in CL users is frequently caused by *P. aeruginosa, *as reported by investigators who have isolated identical organisms from the cornea, the CL and its case, and storage solutions. These studies have focused on refining CL care practices to reduce infections.

CLs are cleaned and disinfected using multipurpose disinfection solutions. The impact of these solutions might decrease when there is increased resistance to disinfection.^[63]^ *In vitro* experiments conducted by Rad et al demonstrated that a suspension of zinc NPs at 250 ppm concentration effectively reduced and inhibited the growth of both gram-positive and gram-negative microorganisms. These results showed that zinc NPs may have applications for lens cleaning and storage solutions due to their antibacterial properties.^[53]^ Another recent study found that low concentrations of silver NPs can reduce the adhesion of *Acanthamoeba* trophozoite to the CL surface and, thus, can be an active ingredient in CL solutions to reduce chances of AK.^[40]^

### Safety and toxicity of nanomaterials

Nanomaterials are increasingly used in ophthalmic drug delivery in response to their potential for targeted drug delivery and improved patient safety. These nanomaterials have unique physicochemical properties, which interact with the biological system. Various nanoformulations are available in the market, but there is a need for further investigations to evaluate the safety, efficacy, and toxicity of these nanomaterials for ophthalmic applications.^[61]^

The toxicity of designed NPs is evaluated using a variety of methods, including *in vitro* experiments, to save time and reduce costs. Tetrazolium reduction assays, lactate dehydrogenase (LDH) assays, immunohistochemical biomarkers for apoptosis, and comet assays for genotoxicity are commonly used methods to measure cell viability. Also, electron microscopy is used to internalize NPs into the cells. Furthermore, compounds such as 3-(4, 5-dimethylthiazol-2-yl)-2,5-diphenyltetrazolium bromide (MTT) and 2-(4-iodophenyl)-3-(4-nitrophenyl)-5-(2,4-disulfophenyl)-2H-tetrazolium, monosodium salt (WST-1), dimethylthiazol-carboxymethoxyphenyl-sulfophenyl-tetrazolium (MTS), and methoxynitrosulfophenyl-tetrazolium carboxanilide (XTT) assay are utilized to identify live cells. MTT is a chemical that easily enters live eukaryotic cells, reduced into purple-colored formazan by mitochondrial succinate dehydrogenase. The MTT tetrazolium test is frequently used to assess cell toxicity, since it involves incubating the reagent with cell cultures and detecting the results based on colorimetric or fluorescence changes. The specific physicochemical features of NPs, such as carbon NPs, might interfere with assay components and produce inaccurate results, leading to varied findings. Furthermore, NPs can induce the formation of reactive oxygen species, which can disrupt mitochondrial enzymes.^[46]^

The cytotoxicity of many NPs made from silica, iron oxide, titanium oxide, and zinc oxide has been assessed using the LDH test. After cellular necrosis, a substantial amount of LDH is released from the cytosol. LDH activity is estimated by a coupled enzymatic reaction. LDH oxidizes lactate to pyruvate, which then interacts with iodonitrotetrazolium chloride (INT) to form a water-soluble formazan that is easily detected using colorimetry at 490 nm. Many researchers have raised concerns about the consistency of LDH assay; for instance, according to Kaja et al, low pH greatly reduces LDH activity whereas high pH destabilizes it.^[64]^

**Table 1:** Summary of different nanomaterials utilized in the development of antimicrobial CLs and lens cases.

**Antimicrobial nanoparticles**	**Substrate**	**Method**	** In vivo/In vitro**	**Microorganisms**	**References**
Silver	Nelfilcon	Soaking	*In vitro*	*P. aeruginosa and Staphylococcus aureus*	42
Copper	Nelfilcon	Soaking	*In vitro*	*P. aeruginosa and Staphylococcus aureus*	42
Zinc oxide- chitosan-gallic acid Nano composite	Comfilcon A	Sonochemical approach	*In vitro*	*Staphylococcus aureus*	68
Phomopsidione nanoparticle	Silicone hydrogel contact lens	Soaking	*In vitro*	*S. marcescens, P. aeruginosa*	69
Copper and poly (carboxylbetaine-co-dopamine methacrylamide)	Pristine	Soaking	*in vitro*	*E.coli, P. aeruginosa, S. aureus, C. albicans*	55
Phytomolecules-coated zinc oxide	Methafilcon A	Adsorption	*In vitro*	*S. aureus, E. coli, and P. aeruginosa*	51
Silver nanoparticles tagged with glycine (GlyH), urea (U), and salicylic acid (SalH2)	2-Hydroxyethyl-methacrylate	Impregnation	*In vitro*	*S. aureus, S. epidermidis, and P. aeruginosa*	62
Phytomolecule-coated silver nanoparticle	Polymeric hydrogel discs	Impregnation	*In vitro*	*P. aeruginosa, E. coli, S. epidermidis, and S. aureus*	47
Ketotifen-loaded gold	Silicon	Impregnation	*In vivo*	White New Zealand rabbits	70
Zwitterionic silver	Poly (2-hydroxyethyl methacrylate) (pHEMA)	Surface immobilization	*In vitro*	*P. aeruginosa, E. coli, S. epidermidis, and S. aureus*	71

Evaluating the toxicity of NPs is difficult due to their distinct physicochemical characteristics, which can disrupt standard testing methods and lead to inconsistent outcomes in toxicological studies. Inflammatory indicators in cell culture, such as chemokine IL-8, TNF-alpha, and IL-6, are found using ELISA. However, because of the special characteristic properties of NPs like size, shape, surface charge, and reactivity, cytokines could obstruct enzymatic immunoassays. Toxicity testing frequently involves cell lines with various pathophysiological properties. Nanomaterials' toxicity is examined through toxicological research, although the results are sometimes contradictory and conflicting.^[10]^ Human exposure to nanomaterials is unavoidable since they are employed in different fields. Therefore, it is recommended to adopt *in vivo* toxicological models that target critical organ systems to evaluate the associated adverse effects and fill the knowledge gap.^[61]^

### Challenges in nano-based strategies for management of microbial keratitis

Clinical translation of several of these drugs has been hampered by challenges in scaling up the nano-based drug delivery systems from the laboratory to large-scale industrial production. For example, it has been reported that various NP properties, particularly their physicochemical characteristics, are subject to change with increased size of nano-based delivery systems. This is also the case for NPs prepared using low-energy techniques like phase inversion temperature, phase inversion composition, and emulsion inversion point methods.^[65]^ Consequently, a number of high-energy techniques have been developed to address the limitations of such low-energy procedures. However, it has been noted that formulating NPs via high-energy techniques, such as ultrasonication and hot homogenization, causes recoalescence and thus renders the system thermodynamically unstable.^[66]^

Furthermore, research indicates that different techniques of creating these nano-based systems typically entail difficult, multi-step processes. Additionally, these operations lack appropriate consistency and repeatability. Therefore, producing nano-based systems is extremely difficult as it leads to issues like batch-to-batch variability and dispersion stability, which further complicates quality control. Particle size and percent yield have been shown to be significantly affected by small changes in a few process parameters. These factors have been shown to have a significant impact on the rate of drug release, encapsulation efficiency, and, ultimately, system performance, because they may alter the pharmacokinetic and pharmacological characteristics of the active components.^[67]^ Novel materials are continuously being identified and utilized in the development of drug delivery systems based on nanotechnology. Nevertheless, it is highly complicated to accurately assess and characterize these systems, which may restrict their application in clinical settings.^[66]^ Compared to their macro size counterparts, nano-based systems are completely different due to their unique particle size. Therefore, a major obstacle to the clinical approval of these formulations has been the inability to accurately ascertain the safety profiles of these systems over time.^[67]^ Because these systems have to be nontoxic and biocompatible with the ocular system, their safety evaluation is crucial. Similarly, it is imperative to ensure that they are quickly digested and do not build up in the eye. The inability to adequately support the biosafety and nontoxicity of the nanosystems, together with their complexity, has hindered their seamless translation from preclinical to clinical studies. Despite obstacles in effectively translating nanotech-based drug carriers for ophthalmology into clinical practice, there is potential for the approval of several nanotechnology products in the near future.^[27]^

##  SUMMARY

This review substantiates that MK can cause corneal scarring, perforation, and ultimately blindness. Wearing CLs is one of the most common predisposing factors for this ocular condition. Presence of pathogenic bacteria, fungi, parasites, and viruses can lead to corneal epithelial disorders, corneal tissue degradation, and even vision loss. Effective diagnosis, treatment, and prevention of MK depend on understanding its prevalence, variety of microbial agents, and predisposing factors. This illness has different demographics and a microbiological profile, with numerous reports published worldwide. Existing treatments for MK have major limitations, including inefficient pathogen recognition, low bioavailability due to traditional drug delivery systems, and the inability of conventional antibiotics to treat multiresistant bacteria.

Owing to their diverse sizes and chemical, physical, optical, and sensing properties, nanomaterials can modify pharmaceuticals, function as drug carriers, or act as therapeutic agents themselves. The introduction of nanomaterials has led to improvements in treating ocular bacterial infections. For example, nanofluorescent probes are able to optically identify MK by analyzing the eye's external surface and transparency in situ. Loading pharmaceuticals onto nanomaterials enhances penetration and adhesion of the drug to the cornea. They offer an antibiotic delivery system with a tailored release schedule. Over the past few decades, antimicrobial approaches for CLs have been explored using silver metal-impregnated lenses and cases. However, toxicity and high cost limit their application. Other metals like copper and zinc nanocoatings may be promising, but in vivo results are scarce.

Systemic administration of NPs still faces several challenges, including toxicology after both short- and long-term exposure, NP interactions with cells, tissues, and organs, determining optimal dosage, selecting acceptable delivery routes, and others. Extensive clinical trials are required before NPs can be administered to patients in order to find the optimal dosage, minimize adverse effects, and achieve continual improvement, thereby reducing treatment time and personalizing the therapy approach. In treating an infection, it is important to detect the type of infection before administering medication. However, few nanomaterials can at the same time prevent, diagnose, and deliver drugs for treatment of ocular diseases like MK. An integrated, versatile nanotherapeutic platform for ocular diseases such as MK will offer ongoing advancements, shorter intervention time, and personalized treatment.

##  Financial Support and Sponsorship

None.

##  Conflicts of Interest

None.
